# Using iterative random forest to find geospatial environmental and Sociodemographic predictors of suicide attempts

**DOI:** 10.3389/fpsyt.2023.1178633

**Published:** 2023-08-01

**Authors:** Mirko Pavicic, Angelica M. Walker, Kyle A. Sullivan, John Lagergren, Ashley Cliff, Jonathon Romero, Jared Streich, Michael R. Garvin, John Pestian, Benjamin McMahon, David W. Oslin, Jean C. Beckham, Nathan A. Kimbrel, Daniel A. Jacobson

**Affiliations:** ^1^Oak Ridge National Laboratory, Computational and Predictive Biology, Oak Ridge, TN, United States; ^2^The Bredesen Center for Interdisciplinary Research and Graduate Education, University of Tennessee Knoxville, Knoxville, TN, United States; ^3^Cincinnati Children's Hospital Medical Center, University of Cincinnati, Cincinnati, OH, United States; ^4^Theoretical Biology and Biophysics, Los Alamos National Laboratory, Los Alamos, NM, United States; ^5^VISN 4 Mental Illness Research, Education, and Clinical Center, Center of Excellence, Corporal Michael J. Crescenz VA Medical Center, Philadelphia, PA, United States; ^6^Department of Psychiatry, Perelman School of Medicine, University of Pennsylvania, Philadelphia, PA, United States; ^7^Durham Veterans Affairs Health Care System, Durham, NC, United States; ^8^VA Mid-Atlantic Mental Illness, Research, Education, and Clinical Center, Seattle, WA, United States; ^9^Department of Psychiatry and Behavioral Sciences, Duke University Medical Center, Durham, NC, United States; ^10^Duke University School of Medicine, Duke University, Durham, NC, United States; ^11^VA Health Services Research and Development Center of Innovation to Accelerate Discovery and Practice Transformation, Durham, NC, United States

**Keywords:** suicide prevention, explainable artificial intelligence, geospatial analysis, public health, veterans’ health, firearms, alcohol misuse

## Abstract

**Introduction:**

Despite a recent global decrease in suicide rates, death by suicide has increased in the United States. It is therefore imperative to identify the risk factors associated with suicide attempts to combat this growing epidemic. In this study, we aim to identify potential risk factors of suicide attempt using geospatial features in an Artificial intelligence framework.

**Methods:**

We use iterative Random Forest, an explainable artificial intelligence method, to predict suicide attempts using data from the Million Veteran Program. This cohort incorporated 405,540 patients with 391,409 controls and 14,131 attempts. Our predictive model incorporates multiple climatic features at ZIP-code-level geospatial resolution. We additionally consider demographic features from the American Community Survey as well as the number of firearms and alcohol vendors per 10,000 people to assess the contributions of proximal environment, access to means, and restraint decrease to suicide attempts. In total 1,784 features were included in the predictive model.

**Results:**

Our results show that geographic areas with higher concentrations of married males living with spouses are predictive of lower rates of suicide attempts, whereas geographic areas where males are more likely to live alone and to rent housing are predictive of higher rates of suicide attempts. We also identified climatic features that were associated with suicide attempt risk by age group. Additionally, we observed that firearms and alcohol vendors were associated with increased risk for suicide attempts irrespective of the age group examined, but that their effects were small in comparison to the top features.

**Discussion:**

Taken together, our findings highlight the importance of social determinants and environmental factors in understanding suicide risk among veterans.

## 1. Introduction

Suicide rates in the United States (U.S.) have increased in recent years despite these rates declining globally (1). According to the biopsychosocial model of suicide risks, there are distal, developmental, and proximal factors that affect the probability of suicide attempt (2). Distal factors are related to familial and genetic predisposition and early-life adversity. Developmental factors include personality traits associated with suicidal behavior, cognitive deficits, and chronic substance misuse. Proximal factors include but are not limited to psychiatric, psychological, socioeconomic, and environmental factors. Several studies have found associations between demographic factors and suicide such as age, ethnicity, socioeconomic status, marital status, religion, etc. (3). The impact of climate on suicidal behavior is significant, although the relationship between climate and suicide is complex and not yet fully understood. One possible explanation for how climate could affect suicidal behavior is through seasonal changes. Research has shown a pattern of increased suicide attempts and deaths during the spring and early summer months, indicating a seasonality in such events (4, 5). Sunlight and temperature are among the most relevant climatic features associated with this seasonality, as they may directly influence various mood disorders related to suicide risk (6–9). While several studies have attempted to link other climatic features to suicide risk, their findings have been contradictory or inconclusive (10–15). Interestingly, the majority of these studies have been conducted using extensive geospatial regions. An investigation carried out in Taipei, Taiwan examined suicide mortality at high geospatial resolution using neighborhoods known as “li” as geospatial units (16). The findings of this study revealed a significant geospatial variation in suicide mortality across neighborhoods, indicating that the analysis of aggregated data in broader geographic areas may attenuate predictive signals (16).

Other relevant proximal factors include access to means and substance misuse (17, 18). For example, occupations with access to lethal means are associated with increased risk of death by suicide (19, 20). Moreover, controlling access to lethal means is an effective strategy for decreasing suicide risk (21). In the U.S., death by suicide is the leading cause of violent deaths, and firearms are responsible for approximately half of these deaths (22). Substance misuse also plays an important role in suicide prevention because acute substance intoxication can increase an individual’s disinhibition. For example, a study showed that suicide decedents have an increased risk of alcohol ingestion and intoxication before their death relative to controls (23).

The objective of the present research was to conduct an analysis of climatic and socio-demographic factors that are associated with increased risk for suicide attempts among U.S. veterans using an explainable artificial intelligence (X-AI) model. We were also interested in the relationship of the number of firearms and alcohol vendors per 10,000 people as proxies for access to means and decreased restraint, which were also included in the final model. Together, we identify several novel factors at zip code-level resolution that impact individual-level risk for attempting suicide.

## 2. Materials and methods

### 2.1. Data and data pre-processing

#### 2.1.1. Patient data

**Figure 1:**
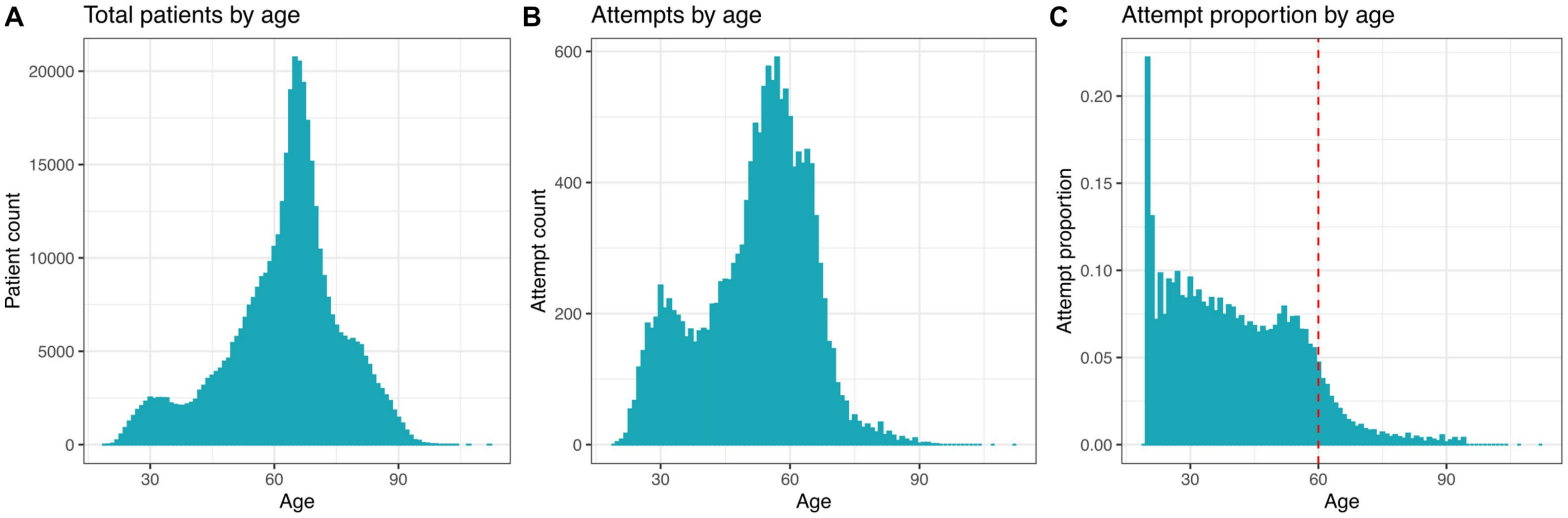
Distribution of suicide attempts by age of 405,540 participating patients. **(A)** Total patient count by age. **(B)** Total suicide attempt count by age. **(C)** Age-specific suicide rate. The red dashed line shows a cutoff to highlight the decreased proportion of suicide attempts in individuals equal to/above 60 years of age.

The cohort and suicide attempt phenotype used in this study were initially described in Kimbrel et al. (24). A total of 405,540 participating patients in this study were enrolled in the U.S. Department of Veterans Affairs’ (VA) Million Veteran Program (MVP). All procedures contributing to this work comply with the ethical standards of the relevant national and institutional committees on human experimentation and with the Helsinki Declaration of 1975, as revised in 2008. All procedures involving human subjects/patients were approved by VA Central Institutional Review Board (cIRB# 18-11) after all subjects provided written and signed informed consent. Race, ethnicity and gender were self-reported. Suicide attempt phenotype was created from electronic health records (EHR) from the VA corporate data warehouse (CDW), using International Classification of Diseases (ICD) diagnostic codes, survey data from the mental health domain, and the Suicide Prevention Application Network (SPAN) data set (25). Veteran participants were considered controls if there was no recorded evidence of them ever attempting suicide or experiencing suicidal thoughts throughout their lives. This determination was based on qualifying ICD codes, reports of suicide behavior, or responses from mental health surveys previously mentioned. It is worth noting that veterans who had a history of having suicidal thoughts but had not attempted suicide were specifically excluded from the current analysis. This was done to guarantee that the control group consisted of individuals who had no prior instances of engaging in or contemplating suicidal thoughts or behaviors. Thus, cases were defined as having a history of one or more suicide attempts (including both fatal and non-fatal), whereas controls were defined has having no history of suicide attempts or ideation. In total, this resulted in 391,409 controls and 14,131 cases. The mean age was 62.4 years for the whole cohort, 63.4 for males and 50.8 years for females. As reflective of the veteran population, this cohort is predominantly male but does include females as well as racial and ethnic minorities. The sex distribution was 8% female and 92% male. This cohort was 73% white, 18% black or African American, 1% American Indian or Alaska Native, 1% Asian, and 7% mixed race, other, missing, or unknown. This is generally close to the proportions of racial groups in the United States in 2021 (78.6% white, 12.2% black/African American, 0.7% American Indian or Alaska Native, 5.6% Asian, 2.8% mixed race) with an overrepresentation of black/African American and an underrepresentation of Asian people. This includes Latinx people spread across those racial categories. The Supplementary Table S1 provides a breakdown of cohort demographics by cases and controls. Most of the suicide attempts in this cohort were concentrated around 60 years of age, likely since this age group is overrepresented among these patients (Figures 1A,B). However, the proportion of attempts grouped by age decreased abruptly after age 60 (Figure 1C). Due to this rapid decrease in attempts after age 60, we analyzed patients greater than 60 years of age separately from those under 60 years of age. The split with patients above or equal to 60 contained a total of 267,447 individuals with 4,231 attempts and 263,216 controls. The split with patients below age 60 was composed of 138,093 individuals with 9,900 suicide attempts and 128,193 controls. Each attempt and control were associated with climatic and socio-demographic features by patient ZIP code. For patients with multiple ZIP codes (less than ~0.6% of the cohort), we used the most recent ZIP code since not all suicide attempts had a corresponding date. The proportion of attempts grouped by age decreased steadily after age 60 (Figure 1). Therefore, we explored the socio-demographic and climatic features that were associated with suicide attempts in patients greater than 60 years of age separately from those under 60 years of age.

#### 2.1.2. Climatic features

The climatic features included two groups: static measurements and monthly measurements. Monthly measurements included longitudinal features such as monthly average precipitation and maximum temperature. There were 12 distinct measurement types recorded each month, totaling 144 features. The 30 static features included features such as elevation and percent urban cover. This led to a total of 174 climatic and weather-related features, mapped to 33,144 ZIP codes across the U.S. (26–32) (Supplementary Table S2).

#### 2.1.3. Socio-demographic features

The socio-demographic features were collected from the 2019 American Community Survey, produced by the United States Census Bureau (33). These 1,606 features were captured using the tidycensus software package in R using 5 years estimate for 2019 (34). These features were normalized to represent a percentage of the total population or age bracket within each ZIP code. We also included two additional features: population density (people per square mile) and the ratio of water to land area, which led to a total of 1,608 demographic features that were mapped to 33,120 ZIP codes across the U.S (Supplementary Table S3).

#### 2.1.4. Alcohol and firearms features

From the Historical Business Database (35) we extracted the number of firearms vendors per 10,000 residents in each ZIP code, averaged across the years 2010 and 2019, and the similarly calculated number of alcohol vendors. This information was included for 31,378 ZIP codes across the U.S (Supplementary Table S4).

### 2.2. Explainable artificial intelligence analysis

In this study, we used iterative Random Forest (iRF) (36, 37), an X-AI algorithm that ranks input features by importance through iterative feature weighting, to associate proximal environmental features with suicide attempts. There are two motivations for our choice of methods: (i) identifying which predictor features explain changes in the output and (ii) scaling to high-performance computing (HPC) systems. iRF has been implemented in C++ to use regression trees and leverages massive parallelism to scale to very large datasets. The model was trained to predict cases and controls, where the predicted values at each leaf node, is the average value of all samples that reached that specific leaf node. Thus, with binary values encoded as case (1) and control (0), iRF identifies the proportional change in outcome as a function of each input feature, thereby ranking features by feature importance.

High pairwise correlations among input features can have a negative impact on the explainability of iRF models. This occurs because when one feature is strongly correlated with others, its importance is divided across the correlated features. As a result, the overall importance of these features in predicting suicide attempts decreases. To identify highly correlated features, we calculated Pearson’s correlation coefficients between all 1,784 features. Through this analysis, we identified feature groups with correlation coefficients equal to or greater than 0.90 in absolute value, and therefore considered as highly correlated. From each feature group of strongly correlated features we selected a representative feature to be included in the iRF model. Thus, this led to the removal of 248 features, reducing the total number of features to 1,536 (Supplementary Table S5). Please refer to Supplementary Table S6 for a comprehensive description of all the features utilized in this study.

### 2.3. iRF k-fold cross validation and accuracy calculation

To obtain accuracy scores for the iRF model, 5-fold cross-validation was used. The cross-validation technique employed in this study was group shuffle split. In this methodology, the dataset was randomly divided into 80% for training and 20% for testing, while incorporating a grouping factor to ensure that samples from the same group were not utilized for both training and testing in the same run. By employing patient zip codes as a grouping factor, we aimed to mitigate geospatial bias in our analysis. To further enhance the robustness of our analysis, we replicated this process five times, resulting in a total of five accuracy estimations for the models. Prediction accuracy was calculated using the average Area Under the Precision Recall curve (AUPRC), where precision was defined as *true positives/true positives + false positives* and recall as *true positives/true positives + false negatives*. Each random forest in the iRF model includes 1,000 trees with a leaf node size of 1,000 patients. We set the number of iRF iterations to five to rank the importance of each feature in predicting suicide attempt. In addition to ranking input features by importance, we next identified the feature-level explainability of our model by determining whether each feature was predictive for or against suicide attempt. To estimate if a feature predicts suicide attempt or controls, the result of each split was averaged using the given feature and mapping those to a linear effect. This provided both the feature effect in the slope of the line and an R^2^ with how closely that related to each of the set of splits. If the slope of the line was positive, then the feature effect size direction was positive, i.e., the value and the feature were positively correlated. If the slope of the line was negative, then the feature effect size direction was negative.

### 2.4. Model selection

**Figure 2:**
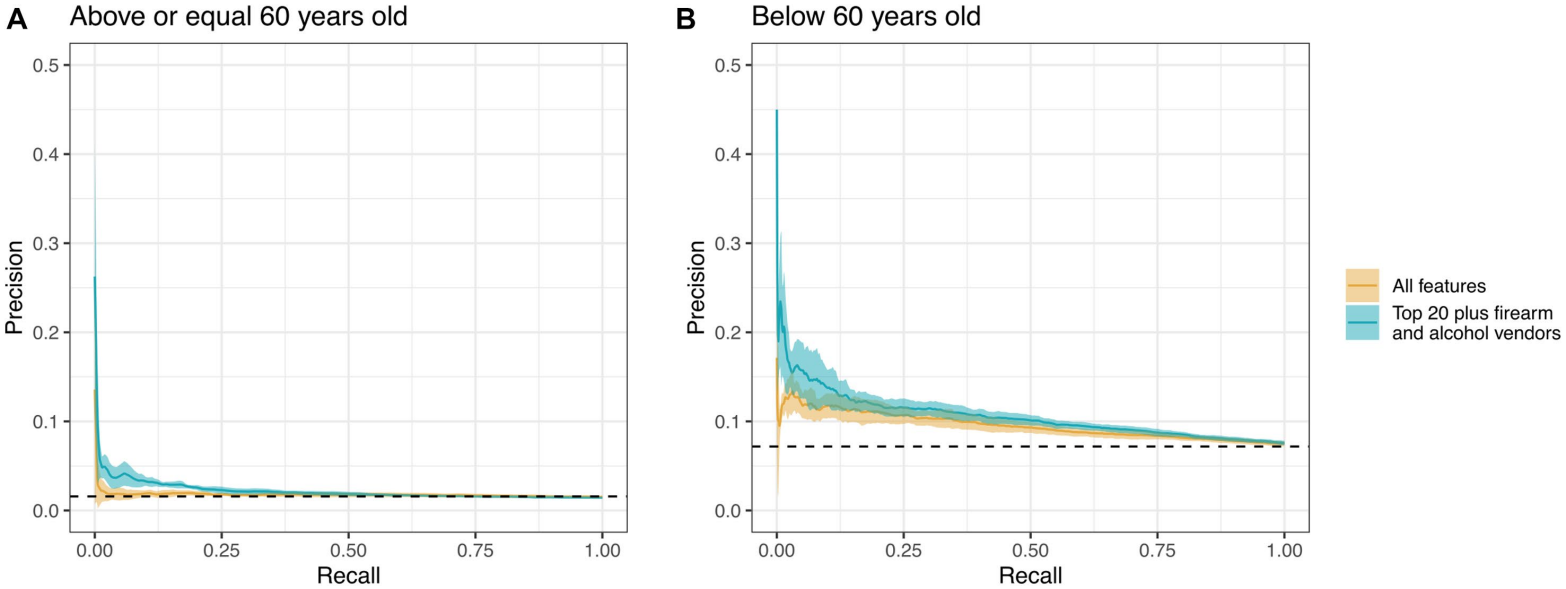
Precision-recall curves for an iRF model using all features (yellow line) vs. using only the top 20 features plus firearm and alcohol vendors per 10,000 residents (teal line). Each line represents the average of 5 runs along their respective 95% confidence intervals. **(A)** Model comprised patients who were 60 years of age or older (*n* = 267,447). **(B)** The model focused exclusively on patients below the age of 60 (*n* = 138,093). The dashed line represents the random chance of correct classification without iRF.

**Figure 3:**
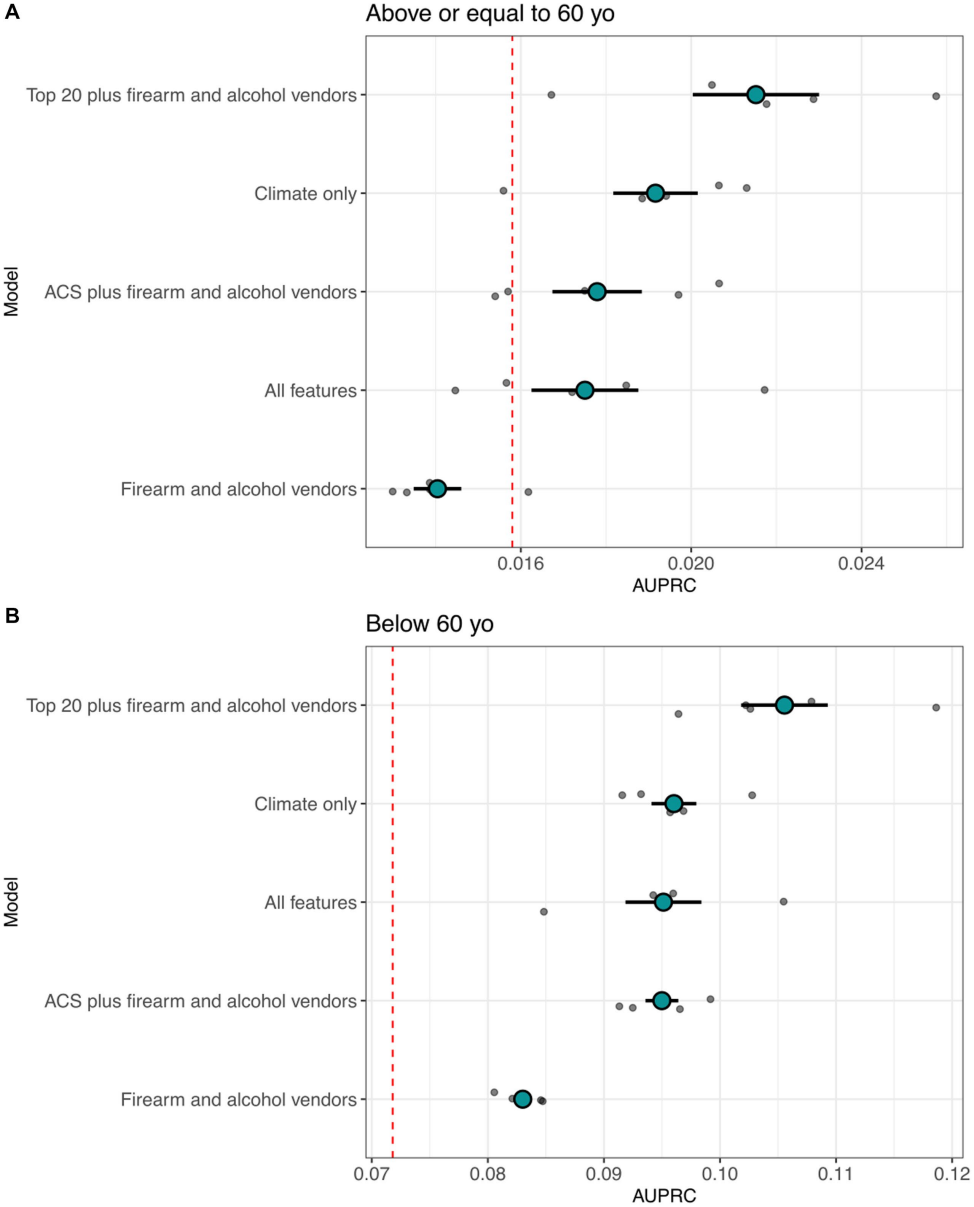
Distribution of the area under the precision recall curves (AUPRC) for suicide attempt predictive capacity using environmental features. **(A)** AUPRC value distributions for equal to/above 60 years of age for iRF cross validation models. **(B)** AUPRC value distributions for below 60 years of age. The red dashed line is the area under the curve of a base model (random chance of correct classification without iRF). The total patient count for the models was 267,447 for individuals aged 60 years or older and 138,093 for those below the age of 60.

Model selection was based on research interest and accuracy gain. Initially, we aimed to address three questions: predicting suicide attempts based on climatic features, demographic features, and access to firearms and alcohol. We used iRF models with k-fold cross-validation for each feature group, and later employed all 1,536 features together to identify the most important predictors for suicide attempts across all feature groups in patients above or equal to 60 years and below 60 years. We then combined the top 20 most important features from the model with all features with alcohol and firearm vendor data per 10,000 residents. The reduced models showed better accuracy than using all features, as indicated by the area under the precision-recall curves (AUPRC) across 5 data splits (Figures 2, 3). Using all features introduced noise and reduced model accuracy and interpretability. Most models demonstrated predictability, with AUPRC values above random chance, especially in the age group below 60 years (red dashed line in Figure 3). Based on these results, we selected the model with the top 20 features, alcohol, and firearm vendors for model explanation.

### 2.5. iRF-LOOP

To show how features or groups of features interconnect each other, we applied iRF-Leave One Out Prediction (iRF-LOOP), which is an extension of the iRF model (36). In this framework, iRF was used to compute all-against-all predictions of each vector of data from all other vectors. The results of this analysis were captured as networks, in which nodes (i.e., features) were connected by an edge if the pair of features were predictive of each other, thereby revealing functional relationships and subgroups within and across data layers. We performed iRF-LOOP using the pre-processed input matrix which consisted of climate, census, and alcohol and firearm business data, with a total of 1,536 features and 31,378 samples or ZIP codes. This analysis created an all-to-all directed feature association network that captured the relationships between data layers. The resulting network was then filtered to the top 1% of edges to capture the most important connections between features.

## 3. Results

### 3.1. Features that were most strongly associated with suicide attempts

**Figure 4:**
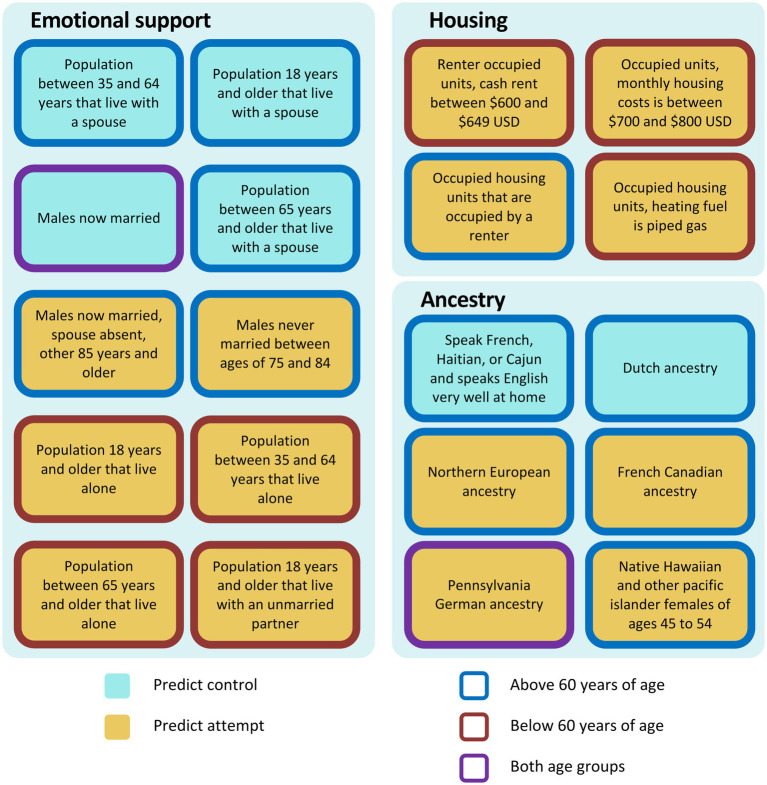
Summary of features found by iRF related to emotional support, housing, and ancestry. Cyan: predict control, yellow: predict attempt, blue border: equal to/above 60 years of age, red border: below 60 years of age, purple border: both age groups.

**Figure 5:**
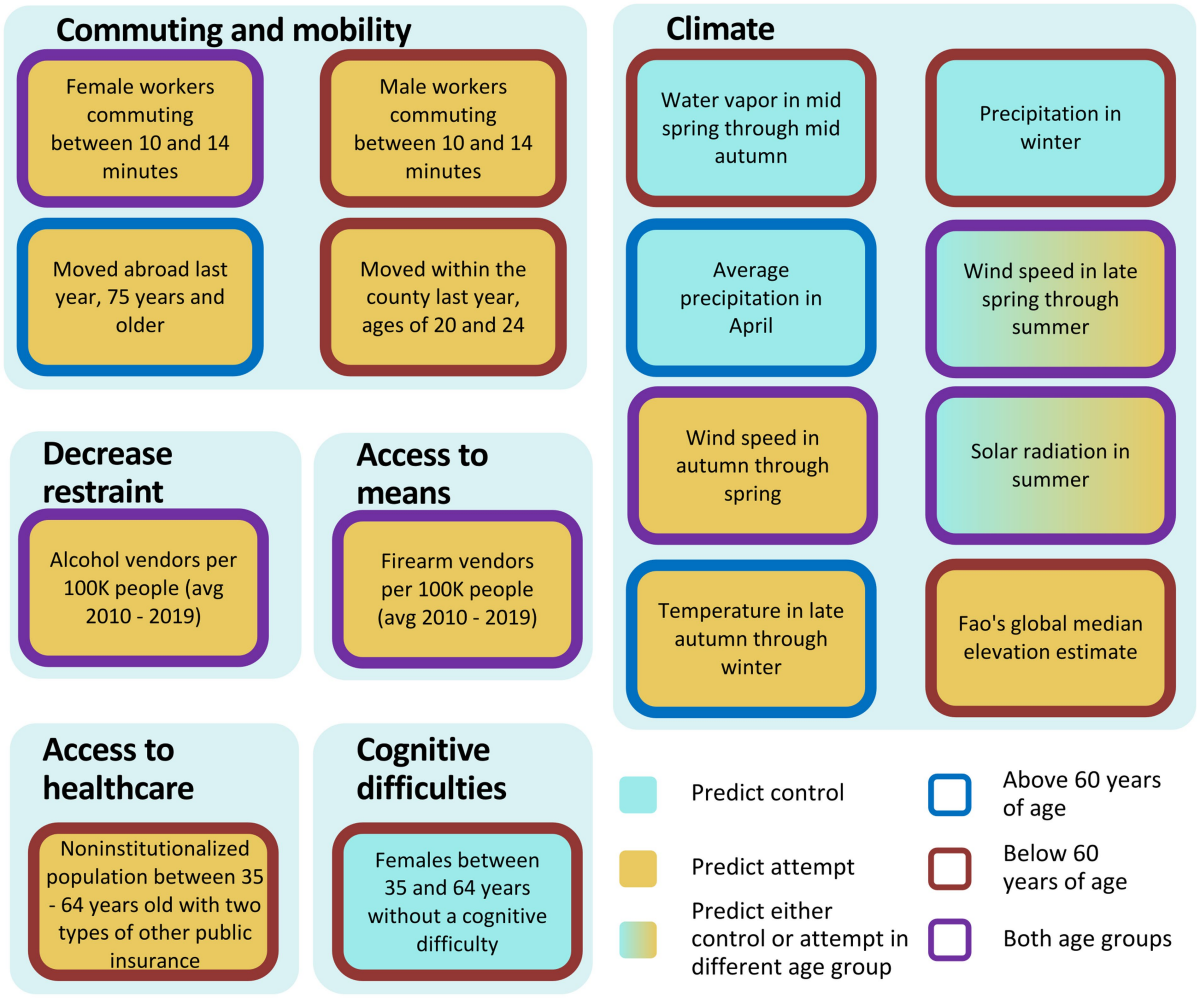
Summary of features found by iRF related to commuting and mobility, climate, decreased restraint, access to means, access to healthcare and cognitive difficulties (proxy for disabilities). Cyan: predict control, yellow: predict attempt, blue border: equal to/above 60 years of age, red border: below 60 years of age, purple border: both age groups.

iRF predictive models can compute the importance of each feature predicting suicide attempt and if they predict either cases or controls. We examined iRF models trained with 1,536 features to identify the 20 most important predictive features in patients below, and above or equal to age 60, plus *firearms and alcohol vendors per 10,000 residents* (Supplementary Figures S1, S2). The resulting features were further analyzed to determine directionality (i.e., if they predict suicide attempts or controls) and can be aggregated in groups related to emotional support, housing, ancestry, commuting and mobility, access to healthcare, cognitive difficulties, access to means, decrease restraint and climate (Figures 4, 5).

In the model that includes patients aged 60 years or older, specific features were found to be associated with either a decreased or increased risk of attempting suicide within the emotional support category. The features *population of 18 years and older that live with a spouse*, *married males* and *household with spouse present between 35–64, and 65 years of age and above* were associated with decreased risk for attempting suicide, whereas *males never married of 75–84 years*, and *married males of 85 years of with spouse absent* were associated with increased risk for attempting suicide (Figure 4). Ancestry also appeared among the top features explaining suicide attempts. *French Canadian*, *Northern European*, and *Pennsylvania German ancestries* were associated with increased risk for suicide attempts, as well as *Native Hawaiian and other Pacific Islander females of ages 45–54* (Figure 4). *Dutch ancestry* and *speaking French (Haitian or Cajun dialects) at home* were associated with reduced risk (Figure 4). In the housing category, *occupied housing units that are occupied by a renter* was predictive of suicide attempts (Figure 4). For commuting and mobility, we observed that *moving abroad last year, 75 years and older*, and *females with a work commute lasting 10–14 min*, were also associated with increased risk (Figure 5). In access to means and decrease restraint groups, we observed that number of firearms and alcohol vendors per 10,000 residents were predictive of suicide attempt (Figure 5). Several climate features were also predictive: *precipitation in April*, *solar radiation in summer* (which represents solar radiation in June, through September), and *wind speed late spring through summer* (which represents wind speed in May through September) were associated with decreased risk, whereas *wind speed in autumn through spring* (which represents wind speed for the rest of the year) and *temperature in late autumn through winter* (which represents average temperature from October through April, minimum temperature from November through March, and maximum temperature from October through March) were associated with increased risk (Figure 5). For representative relationships among features (Pearson >0.9) see Supplementary Table S5.

For the model using only individuals under 60 years of age, the two most important features associated with decreased risk were *married males*, and *females with no cognitive difficulties between 35 and 64 years of age* [which represents a lack of disabilities, including no hearing, vision, ambulatory, or self-care difficulties in females 35–64 years of age (Figures 4, 5)]. Conversely, the features *living alone* and *living with unmarried partner* were associated with increased risk (Figure 4). Similarly, *commuting between 10 and 14 min,* regardless of gender, *monthly housing costs between $700 and $800, cash rent between $600 and $649*, *house heating using gas*, *moved within county,* and *having two types of public insurance* were all associated with increased risk (Figures 4, 5). On the other hand, only *Pennsylvania German ancestry* was associated with increased risk in the ancestry category (Figure 4). Similarly, to the model from patients 60 years of age or above, *number of firearm* and *alcohol vendors per 10,000 inhabitants* were associated with increased risk (Figure 5). Regarding climatic features, *precipitation in winter* (which represents precipitation December through February) and *Water vapor in mid spring through mid autumn* (which represents water vapor from April through October and minimum temperature in May) were associated with reduced risk (Figure 5). Contrary to the first model, in patients under 60 years of age the climatic features *solar radiation in summer*, and both *wind speed in late spring through summer* and *wind speed in autumn through spring* were predictive of individuals with a history of suicide attempt (Figure 5). Additionally, *terrain elevation* was also associated with suicide attempts (Figure 5).

### 3.2. Feature network by iRF-LOOP

**Figure 6:**
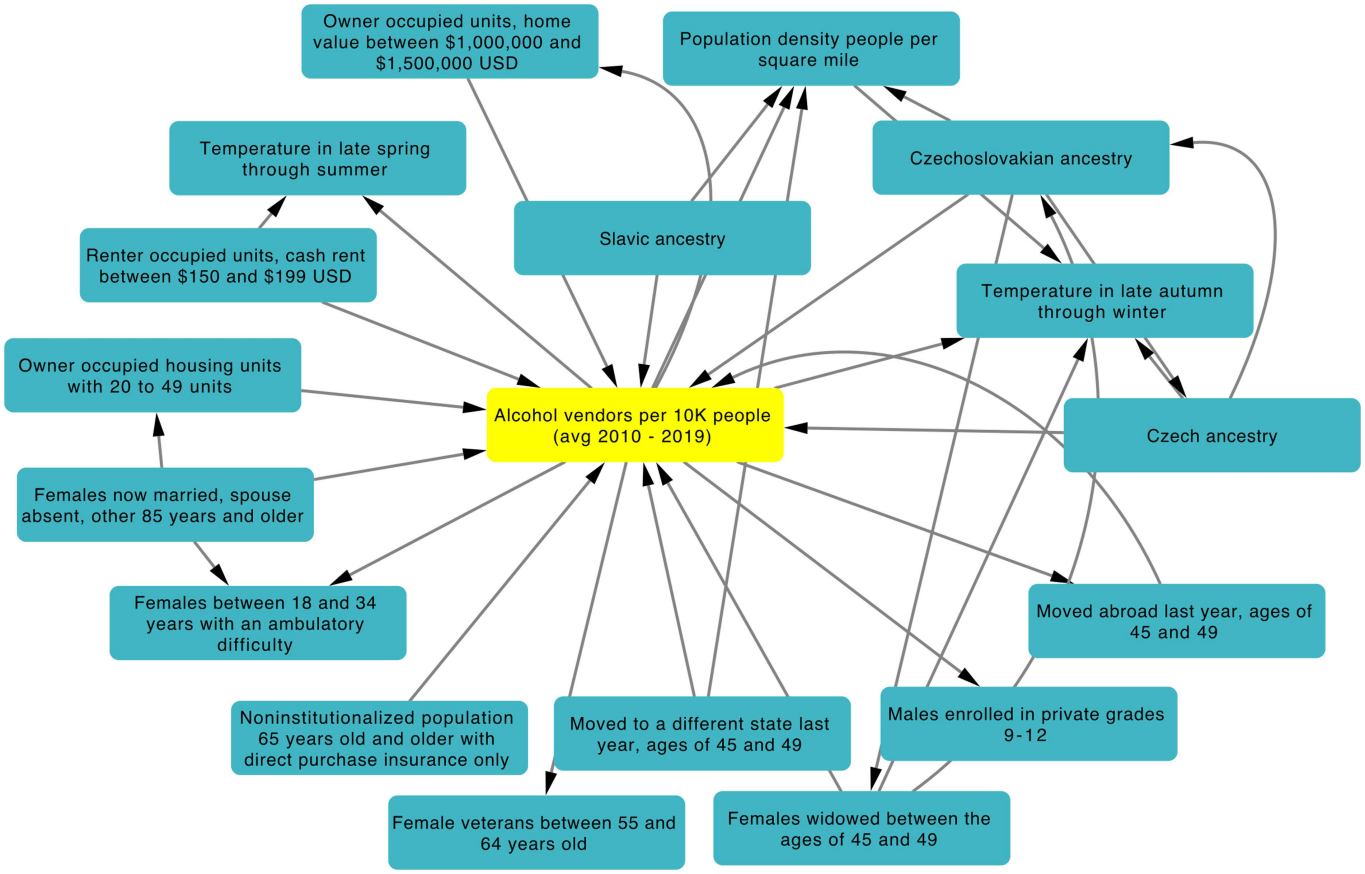
iRF-LOOP Subnetwork showing first neighbors of alcohol vendors per 10,000 people. Only the top 1% of edges weighed by normalized importance are shown. Arrow direction corresponds to one feature predicting another feature.

It is well known that alcohol abuse increases suicide risk (2). Thus, we included the number of alcohol vendors per 10,000 people in our final model. Interestingly, when used in combination with the top 20 features, alcohol vendors per 10,000 people ranked 12th and 20th in feature importance in patients above or equal to 60 and below 60 years of age respectively, even though it ranked 360th (above age of 60) and 74th (below age of 60) in the models using all features (Supplementary Figures S1, S2). This could mean that other features are competing with number of alcohol vendors per 10,000 people within the iRF model to classify suicide attempt and controls. Therefore, we created a feature network using iRF-LOOP to explore how features relate to each other. Figure 6 shows the immediate neighbors of the feature measuring the number of *alcohol vendors per 10,000 people*, where each node in the subnetwork represents a feature and the arrows represent the edges (connections) between features. Edges are weighted by their normalized feature importance value and the arrow direction shows what feature is predicting another one. Several features related to *geographic mobility*, *population density*, *low gross rent with cash*, and *high home value* were observed. Further, the subnetwork shows associations among features including *number of widowed females*, and several *European ancestries*. Regarding climate features, we observed *temperature in late autumn through winter* and *temperature in late spring through summer*, which represents average temperature from May to August, maximum temperature in June, and minimum temperature from June to September.

## 4. Discussion

Most prior suicide prevention studies have focused on a relatively small number of features. Moreover, most have typically relied on individual-level information only (e.g., clinical features) or aggregated data for a given geographical area with low geospatial resolution. In the present study we showed that ZIP code-level data can improve prediction in individuals with a history of suicide attempt greater than random chance, supporting the role of the surrounding environment as proximal/precipitating factors that influence the propensity for an individual to attempt suicide.

Our selection of the iRF model was based on its suitability for our research objectives. In particular, its capacity to efficiently handle large, high-dimensional datasets was crucial for the present study. Furthermore, the model’s feature weighting technique helps to address data overfitting by prioritizing the most informative features while removing the non-relevant ones. This method also enhances the interpretability of the results by generating a ranked list of the most influential features. Lastly, the use of decision trees allowed for the estimation of the directionality of the prediction, enabling a deeper interpretation of the environmental factors contributing to suicidal attempts. Thus, by using this X-AI we were able to screen more than a 1,700 demographic and climatic features to obtain several that appear to potentially protect or increase veterans’ propensity for attempting suicide.

An individual’s environment can have profound effects on their psychological state and subsequent risk of suicide. In fact, it has been well documented that socioeconomic and demographic factors such as poverty, education and population density are related to suicide risk (38–40). In our study we used the electronic health records, demographic and climate data to build a predictive model of suicide attempts in the U.S. veteran population. It is worth highlighting that demographic and climate data are not direct information from the individuals under study, but rather a representation of the environment in which they currently reside. Moreover, in the cohort used here, there was a rapid decline in the number of suicide attempts after the age of 60 (Figure 1), which is in agreement with a surveillance summary from the Center for Disease Control and Prevention, where the highest suicide rates were observed in age group between 35 and 64 years (41). Thus, we divided the population into cohorts below or equal to/above 60 years of age, since age groups may be affected differently by risk factors (42).

Interestingly, the top features can be classified into nine main groups namely: emotional support, housing, ancestry, commuting and mobility, climate, decreased restraint, access to means, cognitive difficulties and access to healthcare (Figures 4, 5). In the emotional support group, the protective effect of marital status on suicide risk is well-documented (43). Studies have shown that married people showed the lowest rate of suicide rates (43–45), whereas divorced or separated persons are twice as likely to commit suicide than married persons, and this effect is stronger in divorced males (46). In our study, for all age groups we observed that living in areas with high proportions of married males and individuals living with a spouse were protective factors against suicide attempts for those 60 years of age or above (similar to the individual-level protective factor of marital status). Conversely, areas with high proportions of individuals living alone and unmarried males were associated with higher rates of suicide attempts (47).

Regarding the commuting and mobility group, commuting time has been associated with depression in a dose responsive manner (48). Our results showed that living in areas with high proportions of individuals with commutes between 10 and 14 min has been associated with increased risk for suicide attempts, irrespective of age. The iRF-LOOP network showed that commuting features are predictive of each other regardless of the commuting time (Supplementary Figure S3). Thus, commuting between 10 and 14 min may be acting as a proxy for commuting in general. We also observed housing-related features for both age groups in relation to suicide attempts. These results are consistent with Lorant et al. (49), who showed that higher education and home ownership status decreases the risk of suicide. Other studies have found that living in rented units increased the risk for suicide in middle-aged males and females (40). This effect might be especially important for females who live in large urban areas (50).

In the ancestry group, we observed that living in areas with a higher proportion of Northern European ancestry was associated with suicide attempts. Although suicide attempts vary widely in Europe, countries of Finno-Ugrian origin show disproportionally higher suicide rates than the rest of Europe, suggesting a genetic cause (51). Voracek et al. (52), tested this hypothesis in the U.S.A. using state-level self-reported ancestry from census data. The study found support for this hypothesis using historical data from 1913–1924 and 1928–1932, but not from 1990–1994. Taken together, these findings encourage an analysis with higher geospatial resolution of the sample (e.g., ZIP or county level) and a genetic characterization of the individuals ancestries, since self-reporting may be inaccurate. We also found that living in a ZIP code with higher proportion of *Native Hawaiian and other pacific islander females between ages 45 to 54* was associated with suicide attempts. These results are consistent with Ji et al. (53) finding, who showed that Asian or Pacific Islander ancestry was a risk factor for suicide in healthcare professionals.

Climate can have a significant impact on suicidal behavior, although the relationship between climate and suicide is complex and not yet fully understood. An explanation of how climate could impact suicidal behavior could be due to seasonal changes. The phenomenon of seasonality in death by suicide has been consistently observed across various studies. A comprehensive literature review encompassing a time span of three decades (1979–2009) revealed a prevailing pattern of increased suicide deaths occurring during the spring and early summer months (4). A subsequent systematic review conducted in 2016 revealed comparable seasonal patterns for suicide attempts, indicating a peak occurrence during the spring and summer months (5). While not all studies in this review identified this seasonal pattern, the majority of them appear to agree with this observation.

Several mood disorders related to suicidal behavior such as depression, bipolar and seasonal affective disorder (SAD) also show seasonality patterns (8). A plausible explanation of this seasonality is the availability of the neurotransmitter serotonin and its receptor, which may be dysregulated in patients with these psychiatric disorders (54–58). However, the exact role of serotonin in major depression is currently under discussion (59, 60). Seasonal variations in serotonin levels have been observed, with winter displaying the lowest levels and summer exhibiting the highest (61). The same study found a direct relationship between serotonin levels and the amount of sunlight on the day of assessment, without significant influence from preceding days. Another study found that individuals exposed to lower solar radiation in the days preceding measurement exhibit reduced postsynaptic serotonin receptor levels (62). Throughout the day, serotonin receptor levels increase while its transporter, which modulates serotonin availability, decreases (63). Intriguingly, longer days coincide with enhanced availability of the serotonin receptor (63). These findings underscore the intricate interplay between sunlight, serotonin, and seasonal fluctuations.

In the present study, we found that solar radiation in summer predicted suicide attempt in patients younger than 60 years. Consistent with this result, a study found that intentional drug overdose deaths in the United States were higher with longer day lengths, which correlates with months with higher solar radiation (64). Conversely, we also found that solar radiation in summer was protective against suicide attempts in patients of 60 years and older. A possible explanation for this variation among different age groups is that older individuals appear to exhibit greater resilience to the seasonal fluctuations of mood changes, as indicated by a self-reported study (65). Nevertheless, the underlying factors contributing to this resilience have yet to be thoroughly explored.

High temperatures are linked to suicide rates (6-8), and they also contribute to an increase in emergency department visits for mental health issues (66). A recent systematic review and meta-analysis revealed that heat negatively affects mental health, potentially by disrupting neurotransmitter balance, causing neuro-inflammation, and disrupting sleep patterns, with the elderly being particularly vulnerable (67). While heat waves typically occur in summer, our study identified that temperatures in late autumn through winter were associated with a higher risk of suicide among patients aged 60 and above. Ajdacic-Gross et al. (68) propose a possible explanation for this inconsistency. Their study, which used moving frames to analyze time series data, found a minor peak of association between suicide risk and summer frames, and a major peak during winter frames. However, it is important to interpret these findings cautiously, as the study was conducted in Switzerland and may not be generalizable to other regions. The biological mechanism underlying these findings has yet to be explored.

Numerous studies have explored the relationship between water vapor (humidity), rainfall, and suicide risk (14, 15). However, the findings either lack evidence of association or yield contradictory results, making it challenging to determine the impact of these features on suicide risk. Our study indicates a potential link between precipitation, water vapor, and reduced suicide risk, but the protective mechanisms behind these features remain inconclusive.

Limited research also exists on the relationship between wind speed and suicide risk. Some studies yield contradictory or inconclusive results (10-14). In our study, we found that wind speed during autumn through spring predicted suicide attempts in both age groups, while wind speed in late spring through summer protected against suicide attempts in patients aged 60 and above. One possible explanation for the predictive nature of wind speed in autumn through spring is its alignment with the tornado season, particularly May, which historically experiences the highest tornado activity. A systematic review revealed that exposure to tornadoes was associated with adverse mental health outcomes (69). While tornadoes have been linked to mental health issues, a separate study found associations between hurricanes, rather than tornadoes, and suicide rates (70).

Although terrain elevation is not inherently a climatic feature, we classified it as such due to its association with various climatic factors that undergo changes alongside it. Our study revealed a positive association between altitude and suicide attempts, aligning with a systematic review that showed a similar trend in 17 out of 19 analyzed studies (70, 71). Hypobaric hypoxia-induced dysregulation of serotonin levels has been proposed as a potential mechanism for increased suicide risk at high altitudes, but further empirical testing is required (72).

Another goal of the present work was to explore decreased restraint and access to means for suicide attempts using the number of alcohol and firearms vendors per 10,000 residents as proxies. Importantly, regardless of age, we observed that living in areas with more alcohol and firearm vendors was associated with increased risk for attempting suicide (albeit less so than several of the climatic and socio-demographic features identified as important). These results provide additional evidence regarding the importance of access to means as one of several risk factors for suicide, especially in the U.S., where a larger proportion of suicides are committed using firearms in comparison to other high-income countries where only 5% use them (22, 73, 74). Our findings also confirm the importance of including alcohol abuse in models of suicide risk (75, 76).

In this study we demonstrated the use of X-AI to explore the impact of more than 1,700 demographic and climatic features on suicide attempt risk with high geospatial resolution. This research provides additional evidence for the role of several demographic and climatic features in suicide attempts and demonstrates the utility of using geospatial features from the area (e.g., neighborhoods/communities) in which patients live within an X-AI framework to improve suicide risk prediction. By focusing solely on sociodemographic and environmental factors, we aimed to determine their distinct influence on suicide risk, regardless of clinical or psychiatric factors. We also aimed to investigate their influence from a public health standpoint. Analyzing these factors can provide valuable insights for developing effective interventions and policies to decrease suicide risk. By identifying the key factors that contribute to suicide risk, researchers and policymakers can prioritize interventions that specifically target these factors. For example, understanding the localized variations in risk factors can help target interventions to specific regions or communities. Suicide prevention measures can be tailored based on the unique sociodemographic and environmental characteristics of different areas, allowing for more effective and efficient allocation of resources. Interventions could include promoting social support networks, providing tailored mental health services, and implementing community-based programs that address the unique needs and challenges of vulnerable populations. Overall, the findings contribute valuable insights to suicide prevention measures by highlighting the role of sociodemographic and environmental factors and emphasizing the need for a holistic, geospatially-informed approach. By integrating these findings into policies and interventions, it is possible to develop more effective strategies for preventing suicide and promoting mental well-being in at-risk populations.

### 4.1. Limitations

The present study had a number of limitations that should be considered when interpreting these findings. First, we utilized a cross-sectional design, and a lifetime suicide attempts variable. Thus, additional work is still needed to examine the degree to which the features identified in the present study might be predictive of future suicide attempts. Second, our findings regarding alcohol and firearms vendors should be interpreted cautiously, as there are several other features (Supplementary Figure S4) that could also potentially explain their association with suicide attempts which should be considered in future work. Third, it is important to note that the cohort examined in this study primarily consisted of males of Caucasian descent, with an average age of about 60. Consequently, caution must be exercised when generalizing these findings to individuals of different racial backgrounds, age groups, and genders. Fourth, while the present study provides clear evidence that zip code-level geospatial features can predict suicide attempt risk better than random chance, the degree to which these features might predict suicide attempts above and beyond well-validated individual-level risk factors (e.g., psychiatric diagnosis) remains unknown. While such work is beyond the scope of the present study, future work is still needed to ascertain if and how geospatial features might interact with individual-level data to predict suicide risk.

## Data availability statement

Suicide attempt data from individual patients is not readily available because this data is considered Protected Health Information by the U.S. Department of Veterans Affairs. All climate data and socio-demographics can be made available upon request.

## Ethics statement

The studies involving human participants were reviewed and approved by VA Central Institutional Review Board (cIRB# 18-11). The patients/participants provided their written informed consent to participate in this study.

## Author contributions

MP, JL, AW, KS, MG, NK, and DJ: conceptualization. MP, JL, AC, JR, and DJ: methodology. MP: validation, investigation, and visualization. MP and AW: formal analysis. MP, AW, and JS: data curation. MP, KS, JS, MG, JP, and NK: data interpretation. MP, JL, and AW: writing of the original draft. MP, JL, KS, JS, MG, BM, DO, JB, NK, and DJ: writing—review and editing. DJ: funding. NK and DJ: supervision. All authors contributed to the article and approved the submitted version.

## Funding

This research is based on data from the Million Veteran Program, Office of Research and Development (ORD), Veterans Health Administration (VHA), and was supported by award #I01CX001729 from the Clinical Science Research and Development (CSR&D) Service of VHA ORD. This work was also supported in part by the joint U.S. Department of Veterans Affairs and US Department of Energy MVP CHAMPION program.

## Conflict of interest

The authors declare that the research was conducted in the absence of any commercial or financial relationships that could be construed as a potential conflict of interest.

## Publisher’s note

All claims expressed in this article are solely those of the authors and do not necessarily represent those of their affiliated organizations, or those of the publisher, the editors and the reviewers. Any product that may be evaluated in this article, or claim that may be made by its manufacturer, is not guaranteed or endorsed by the publisher.

## Author’s disclaimer

This publication does not represent the views of the Department of Veteran Affairs or the United States Government. This manuscript has been co-authored by UT-Battelle, LLC under contract no. DE-AC05-00OR22725 with the U.S. Department of Energy. The United States Government retains and the publisher, by accepting the article for publication, acknowledges that the United States Government retains a nonexclusive, paid-up, irrevocable, world-wide license to publish or reproduce the published form of this manuscript, or allow others to do so, for United States Government purposes. The Department of Energy will provide public access to these results of federally sponsored research in accordance with the DOE Public Access Plan (http://energy.gov/downloads/doe-public-access-plan, last accessed September 16, 2020).
